# Genitourinary

**DOI:** 10.4103/0971-3026.40310

**Published:** 2008-05

**Authors:** B Santh Kumar

A 50-year-old man presented with a history of right testicular pain for one week. On examination, there was tenderness in the right hemiscrotal region. An ultrasound (USG) image of the right testis is shown [Figure 1].

**Figure 1:**
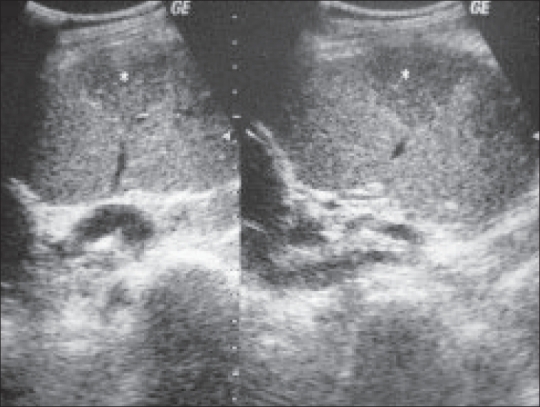
USG of the right testis

## What is the Diagnosis?

### Diagnosis: Segmental Testicular Infarct

**Figure 2:**
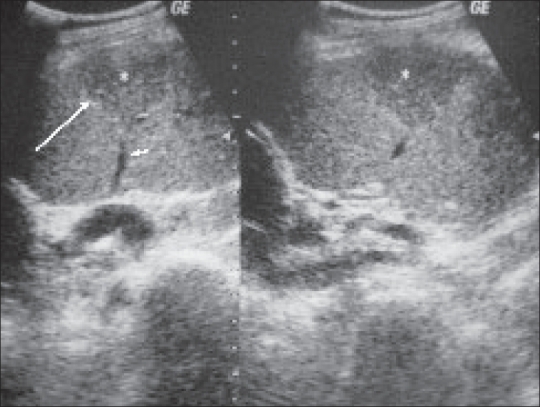
USG of the right testis showing a wedge-shaped lesion (large arrow). A linear hypoechoic vessel is seen terminating at the tip of this wedge-shaped triangular lesion (small arrow)

In this patient, there is a wedge-shaped triangular lesion in the mid-pole of the testis, with a blood vessel ending at the apex of the lesion [Figure 2]. A color Doppler study (not shown) did not reveal any flow. These findings were suggestive of a segmental testicular infarct and, therefore, surgery could be avoided.

Segmental testicular infarction is rare and very few cases have been reported till date.[1,2] It has also been known to occur in neonates.[3] There are various causes described for segmental testicular infarction, which have been summarized by Sriprasad *et al*.[1] It may be idiopathic or may be due to polycythemia, intimal fibroplasia of the spermatic artery, sickle cell disease, hypersensitive angiitis, or trauma. In most patients, the cause is not known, as was the case in our patient.[1,2]

The exact mechanism is also unknown. Reduction in blood flow due to venous thrombosis, in an end-organ such as the testis can cause segmental infarction.[4,5] Segmental testicular infarction presents with acute testicular pain and tenderness.[2] Radiologically, the most important differential diagnosis is from a testicular tumor. Clinically, however, a tumor does not present with acute pain.[1,2,6]

Color Doppler and MRI are the imaging modalities of choice for a precise and correct preoperative diagnosis of segmental testicular infarction.[1,7,8] The pattern of segmental testicular infarction can be heterogeneous or hemorrhagic.[1,5] However, the presence of a triangle-shaped, avascular, intra-testicular lesion on USG or MRI, and enhancement of the surrounding borders on enhanced MRI images, may suggest a diagnosis of segmental testicular infarction and can, therefore, help prevent unnecessary total orchidectomy in these patients.[8]
